# Dynamic nuclear envelope phenotype in rats overexpressing mutated human torsinA protein

**DOI:** 10.1242/bio.032839

**Published:** 2018-05-08

**Authors:** Libo Yu-Taeger, Viktoria Gaiser, Larissa Lotzer, Tina Roenisch, Benedikt Timo Fabry, Janice Stricker-Shaver, Nicolas Casadei, Michael Walter, Martin Schaller, Olaf Riess, Huu Phuc Nguyen, Thomas Ott, Kathrin Grundmann-Hauser

**Affiliations:** 1 Institute for Medical Genetics and Applied Genomics, University of Tuebingen, Calwerstr. 7, 72076 Tuebingen, Germany; 2 Centre for Rare Diseases, University of Tuebingen, Calwerstr. 7, 72076 Tuebingen, Germany; 3 Core Facility Transgenic Animals, University Hospital Tuebingen, Otfried-Mueller-Str. 27, 72076 Tuebingen, Germany; 4 Agilent Technologies, 5301 Stevens Creek Blvd, Santa Clara, CA 95051, USA; 5 Department of Dermatology, University of Tuebingen, Liebermeisterstr. 25, 72076 Tuebingen, Germany

**Keywords:** DYT1 dystonia, TorsinA, Transgenic, Rat, Nuclear envelope, Pathology

## Abstract

A three-base-pair deletion in the human *TOR1A* gene is causative for the most common form of primary dystonia: the early-onset dystonia type 1 (DYT1 dystonia). The pathophysiological consequences of this mutation are still unknown. To study the pathology of the mutant torsinA (TOR1A) protein, we have generated a transgenic rat line that overexpresses the human mutant protein under the control of the human *TOR1A* promoter. This new animal model was phenotyped with several approaches, including behavioral tests and neuropathological analyses. Motor phenotype, cellular and ultrastructural key features of torsinA pathology were found in this new transgenic rat line, supporting that it can be used as a model system for investigating the disease’s development. Analyses of mutant TOR1A protein expression in various brain regions also showed a dynamic expression pattern and a reversible nuclear envelope pathology. These findings suggest the differential vulnerabilities of distinct neuronal subpopulations. Furthermore, the reversibility of the nuclear envelope pathology might be a therapeutic target to treat the disease.

## Abstract (precis)

**Summary:** A novel transgenic rat model displaying dystonia-like phenotypes and dynamic processes in NE pathology can become a useful tool for therapy development for dystonia and other related diseases.

## INTRODUCTION

Early-onset dystonia type 1 (DYT1 dystonia) is an inherited autosomal-dominant disease characterized by involuntary movements due to a dysfunction of the central nervous system (CNS) involving selective regions implicated in movement control (for review see Albanese et al., 2013; Klein, 2014; Ledoux et al., 2013; Tanabe et al., 2009). DYT1 dystonia is caused by a three-base-pair deletion (ΔGAG) in the *TOR1A* gene (Ozelius et al., 1997). Clinical symptoms including twisting movements and abnormal posture are due to involuntary muscle contractions and they manifest predominantly in childhood. Findings from *in vitro* studies, neurochemical analyses, functional imaging and electrophysiological studies in mouse models and patients indicate that dystonic movements are the result of a dysfunction of the CNS motor system (Martella et al., 2009; Niethammer et al., 2011; Tanabe et al., 2009).

The torsinA protein is an ATPase protein associated with different cellular activities (AAA+ proteins) that belongs to a small family of torsin-like proteins (for review see Rose et al., 2015). Studies in recent years have implicated the functions of torsinA in several physiological aspects, including cellular response to stress (Hewett et al., 2003; Kuner et al., 2004; Shashidharan et al., 2005), neurite outgrowth (Ferrari-Toninelli et al., 2004; Hewett et al., 2006), synaptic plasticity (Martella et al., 2009) and dopaminergic transmission (Augood et al., 2004, 2002; Torres et al., 2004), but the native function(s) and the pathophysiological consequences of this mutation are still unclear.

A variety of animal models for DYT1 dystonia have been generated for analyzing the disease and they include both transgenic and gene-targeted mouse models (Dang et al., 2005; Goodchild et al., 2005; Grundmann et al., 2007; Sharma et al., 2005; Shashidharan et al., 2005). It has been reported that homozygous DYT1 knock-in and knock-out animals died shortly after birth (Goodchild et al., 2005) and GAG deletion caused redistribution of torsinA from the endoplasmic reticulum (ER) to the nuclear envelope (NE) that eventually led to NE-pathology (Goodchild and Dauer, 2004; Misbahuddin et al., 2005), abnormal protein interaction (Goodchild and Dauer, 2004, 2005) and formation of inclusion bodies in cell culture (Hewett et al., 2000). Moreover, it has been shown that the pathology of DYT1 dystonia is not restricted to dopaminergic dysfunction of the basal ganglia but also involves other neurotransmitters (e.g. serotonin) and brain regions (e.g. brainstem) (Grundmann et al., 2007).

A more recent study on conditional *TOR1A* mice that either lacked torsinA or expressed mutant torsinA exclusively in the CNS showed that these mice displayed perinuclear abnormalities in discrete sensorimotor regions followed by neurodegeneration and their behavioral impairments emerged with increased age (Liang et al., 2014). This study demonstrated the importance of torsinA in key neuroanatomical structures during postnatal CNS development.

To show that the pathological features previously observed in the dystonia mouse models are attributed solely to the mutant torsinA protein, introducing the mutation in a second rodent species is necessary to prove that these features are not artefacts of a species-specific event. We have hence generated a transgenic rat model over-expressing the human mutant Tg(TOR1A-TOR1A^ΔGAG^)4Olri) (tghΔGAGTorAl4) and wild-type torsinA Tg(TOR1A-TOR1A)11Olri) (tghwtTorAl11) proteins under the control of the endogenous human promoter and its regulatory elements. We have previously shown that over-expression of mutated torsinA led to behavioral abnormalities and NE pathology (Grundmann et al., 2012). Unexpectedly, this rat line (tghΔGAGTorAl4) also developed diabetes mellitus.

To further investigate the physiological function(s) of torsinA and the impacts of its mutation *in vivo*, we have characterized another transgenic rat line (line 8, tghΔGAGTorAl8), generated by using the same methodology (Grundmann et al., 2012), with detailed characterization of motor function as well as analyses of neuropathological changes in selective brain regions by immunohistochemistry and electron microscopy. As torsinA has been demonstrated to have a role during postnatal CNS development (Liang et al., 2014), we have examined the brains of the rats at different ages [postnatal day (P) 0–30, 2 and 20 months of age] so as to assess the onset and the development of region-specific and age-dependent neuropathological features when the disease progressed.

## RESULTS

### Behavioral phenotypes

To examine whether phenotypic abnormalities result from expression of mutated torsinA or merely represent artefacts, and to establish an appropriate model system for further investigation, the previously characterized tghΔGAGTorAl4 line (line 4), which developed diabetes mellitus by the age of 11 months, was compared to the second transgenic line, namely tghΔGAGTorAl8 (line 8).

**Fig. 1.**
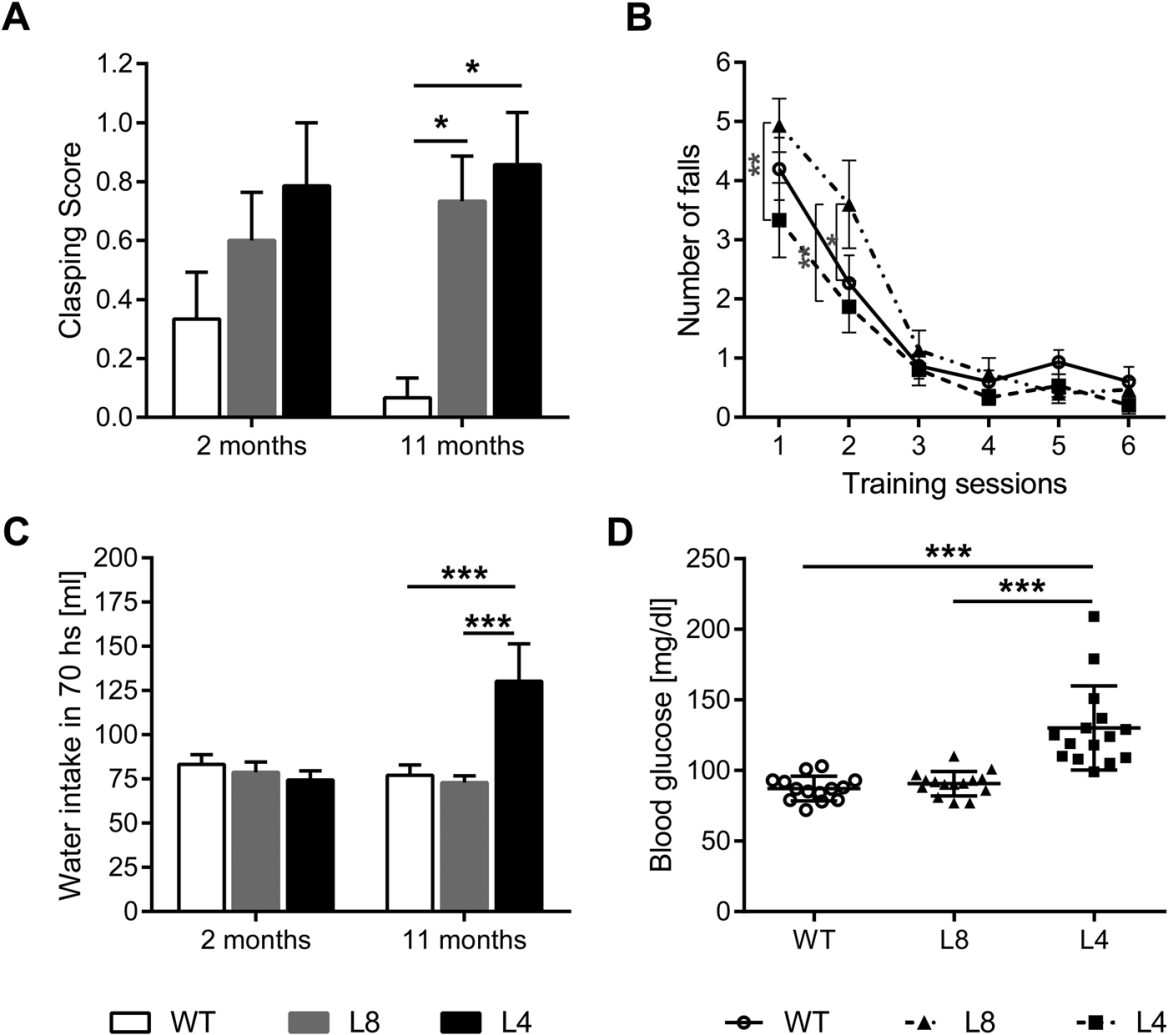
**Behavioral phenotype of tghΔGAGTorAl8 rats.** (A) Hind limb clasping phenotype of tghΔGAGTorAl8 rats compared with tghΔGAGTorAl4 and non-transgenic littermates at younger and older ages. Both tghΔGAGTorAl8 and tghΔGAGTorAl4 rats showed increased hind limb clasping posture compared with non-transgenic controls at 11 months of age when suspended by tail. (B) Number of falls during the first six rotarod training sessions at 2 months of age. TghΔGAGTorAl8 rats showed a worse performance compared with tghΔGAGTorAl4 rats at training session 1 and when compared with both tghΔGAGTorAl4 rats and non-transgenic controls at training session 2. TghΔGAGTorAl8 rats and non-transgenic controls showed comparable water consumption at 2 and 11 months of age (C) as well as a similar blood glucose level at 9 months of age (D), while both water consumption (C) and blood glucose (D) are significantly increased in tghΔGAGTorAl4 rats compared with other two groups at 11 and 9 months of age respectively. For all experiments, the same cohort of rats were used: *n*=15 for each group. Data were analyzed by one-way ANOVA or two-way ANOVA with Tukey's or Dunnett’s post-hoc test. Data are presented as mean±s.e.m. *P<0.05; **P<0.01; ***P<0.0001.

Hind limb clasping was examined in torsinA transgenic rats of lines 4 and 8 as well as in non-transgenic controls at 2 and 11 months of age. Transgenic rats of both lines showed a trend towards hind limb clasping at 2 months of age as indicated by their higher scores and they reached significant levels at 11 months of age in both lines [line 4 (*P*<0.01) and line 8 (*P*<0.05)] when compared to non-transgenic controls. No difference was found between tghΔGAGTorAl4 and tghΔGAGTorAl8 rats (Tukey's multiple comparison test) (Fig. 1A).

Previously we reported that tghΔGAGTorAl4 rats displayed an abnormal motor learning behavior when performing the rotarod test (Grundmann et al., 2012). In this study, the same protocol was used to compare the motor learning ability and motor coordination of tghΔGAGTorAl4, tghΔGAGTorAl8 and non-transgenic control rats. During training sessions, all three groups of rats reached a performance plateau in the third session. No main effect of genotype or interaction between genotype and learning session was seen. However, the rotarod performance of 2-month-old tghΔGAGTorAl8 was significantly worse than their non-transgenic controls on the second training sessions (Fig. 1B, *F*=3.35, *P*<0.05), which is consistent with our previous analysis that transgenic rats expressing mutant torsinA are impaired in motor behavior (Grundmann et al., 2012). No differences were found in the rotarod test sessions between rats of line 8 and non-transgenic control rats. This is in contrast to line 4 rats, which showed a significantly higher latency to fall at the age of 11 months that was possibly due to decreased body weight (Fig. S1). Regression analyses of the body weight and the latency to fall show that it is generally true for a reduced body weight to result in an increased latency time to fall independent of the genotype (Fig. S2). No differences among genotypes were observed in the average latency of the beam walking assay (Fig. S3).

To study the behavior of the various rat lines as compared to their wild-type counterparts, animals were systematically analyzed in the automated PhenoMaster home cages at the age of 2 and 11 months. Up to 11 months of age, no differences could be found among line 4, line 8 and non-transgenic rats with respect to parameters including distance moved, ambulatory activity and rearing activity (Fig. S4).

### Diabetes was not detectable in tghΔGAGTorAl8 rats till 11 months of age

Since the tghΔGAGTorAl4 rats developed diabetes with age (Grundmann et al., 2012), it was possible that tghΔGAGTorAl8 rats would have a similar susceptibility to developing diabetes. Hence, we analyzed their drinking behavior using automated measurement. Increased level of water consumption was only found in line 4 when compared with line 8 and non-transgenic rats at 11 months of age, while the drinking behavior of line 8 rats showed the same level as non-transgenic controls at both times of measurement (Fig. 1C), suggesting that there was no late onset glucose intolerance in line 8. In addition, blood glucose levels were analyzed, and they were found to have increased by the age of 11 months in transgenic rat line 4 (*P*<0.001), but not in rat line 8 (Fig. 1D). In contrast to line 4, the total body weight and the increase in body weight of line 8 rats were similar to wild-type animals up to the age of 11 months (Fig. S1B).

### Mutant torsinA in the brain of tghΔGAGTorAl8 rats has a dynamic temporal and spatial protein expression pattern

**Fig. 2.**
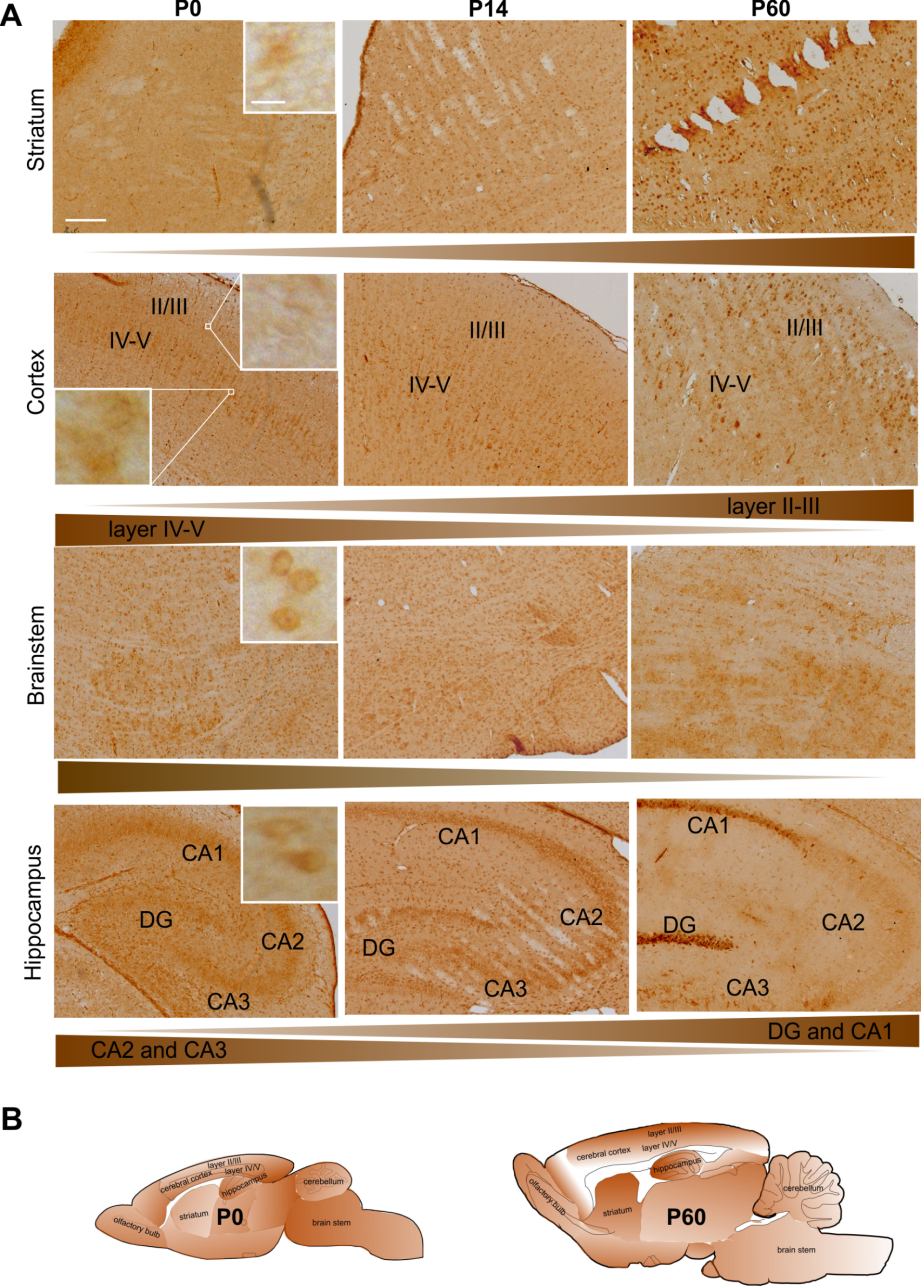
**Temporal and spatial protein expression pattern of torsinA in tghΔGAGTorAl8 rats.** Sagittal brain sections of tghΔGAGTorAl8 rats at P0, P14 and P60 were analyzed by immunohistochemical staining using anti-torsinA. (A) Representative images of brain regions showed that protein expression level of torsinA increased with age in the striatum, cortical layers II-III, and the DG and CA1 zones of the hippocampus, while the brainstem, the cortical layers IV-V and the CA2 and CA3 zones showed a decreased protein expression level of torsinA. Scale bar: 200 µm; magnified inset, scale bar: 10 µm. (B) Schematic overview of torsinA distribution at P0 and P60. The intensity of brown color represents the abundance of torsinA protein.

Expression of torsinA in rodents and humans is temporarily regulated during embryonic and postnatal development. In rodents, torsinA expression peaks at P0 and declines postnatally (Vasudevan et al., 2006; Xiao et al., 2004). In order to determine the expression of torsinA in this model system, we prepared brains from early postnatal stages to further investigate the expression pattern and the onset of NE pathology in tghΔGAGTorA transgenic rat brains compared with non-transgenic wild-type controls. Temporal and spatial protein expression pattern of transgenic human torsinA in tghΔGAGTorAl8 rats was assessed using immunohistochemistry staining at P0, P14 and P60. Detailed expression analysis of brain sections from rat line 8 at P0 showed the most abundant protein expression in the brainstem, the cerebellum (data not shown) and the hippocampus. Less abundant mutant torsinA was present in layers IV-VI of the cerebral cortex, the olfactory bulb (data not shown), and the hypothalamus (data not shown). Only slight torsinA expression was detected in the striatum and layers II-III of the cerebral cortex (Fig. 2). Notably, from P0 to P60, the protein expression level of mutant torsinA increased in some brain areas, especially in the striatum and the cortical layers II-III, while it decreased in the cortical layers IV-VI and the brainstem. At P60, the most abundant torsinA expression was found in the striatum and hippocampus [CA1 and dentate gyrus (DG) zones] as well as the cortical layers II-III, as shown by the intensely stained cell soma and proximal dendrites of some pyramidal cells. In contrast, lower intensity of staining was observed in the brainstem compared with the other brain areas and the same pattern was observed at P0 (Fig. 2A). In cortical layers IV-V, protein expression was detected in fewer cells although with higher staining intensity when compared to P0. The temporal and spatial protein expression pattern is summarized in Fig. 2B.

### Mislocalization of mutant torsinA to the NE and formation of inclusion bodies was found in early age of tghΔGAGTorAl8 rats

**Fig. 3.**
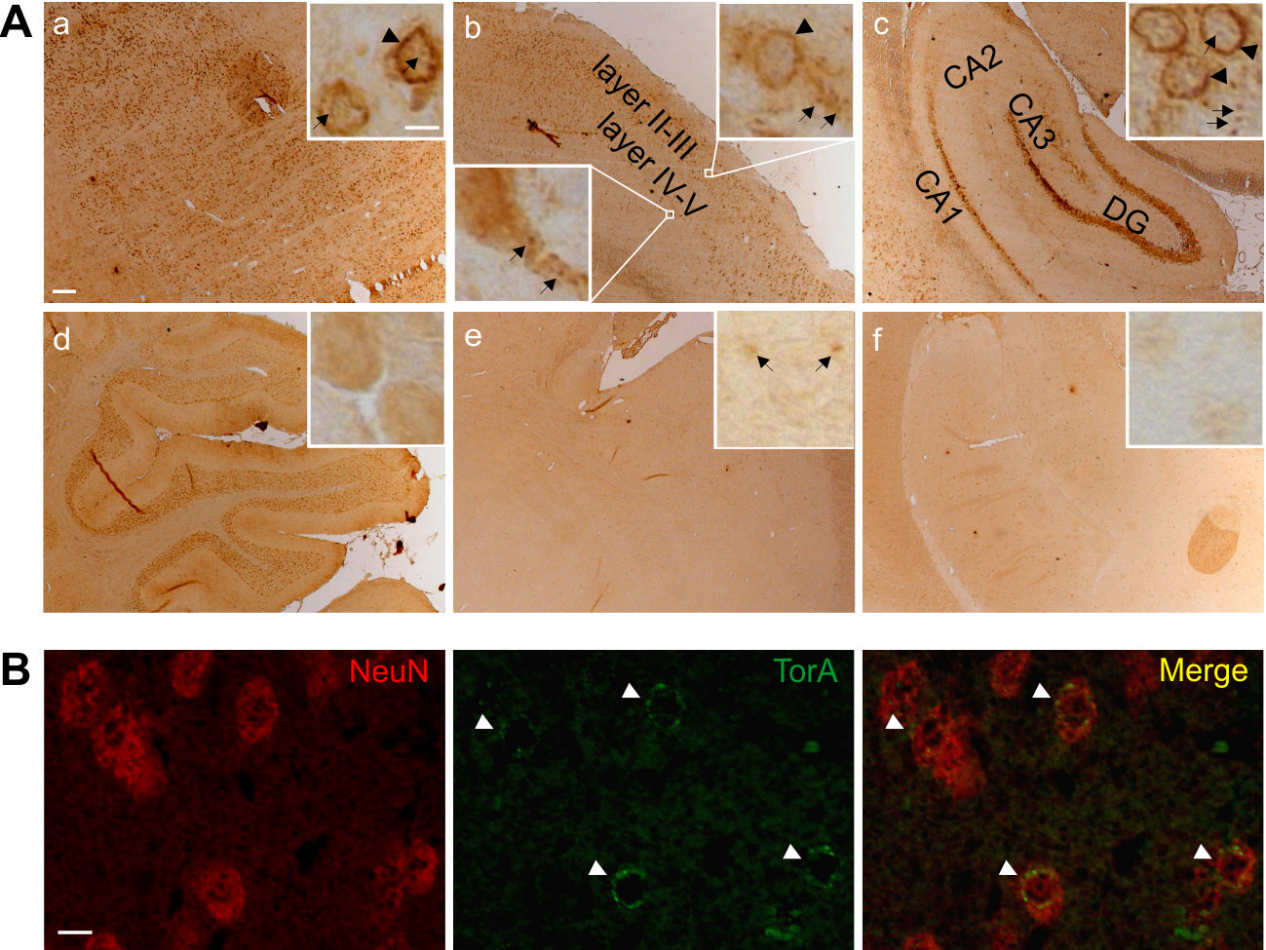
**Brain region- and cell type-selective aggregation (NE-accumulation and inclusion bodies) of torsinA in tghΔGAGTorAl8 rats at 2 months of age.** (A) The anatomical distribution of torsinA aggregation at 2 months of age. Brain sections of tghΔGAGTorAl8 rats at 2 months of age were stained by anti-torsinA. The most abundant NE-accumulation (arrowheads) appeared in the striatum (a), the cortical layers II-III (b), and the DG and CA1 zones of the hippocampus (c). Compared with cortical layers II-III, layers IV-V exhibited fewer cells with torsinA NE-accumulation (c). Intranuclear inclusion bodies in the striatum (a), neuropil inclusion bodies in the cortex (b) and both types of inclusions in the hippocampus (c) are shown in the magnified inset (arrows). In the cerebellum (d), torsinA was homogenously distributed in the cytoplasm with no torsinA aggregation. The striatum, the brain area with the most abundant torsinA aggregation in the transgenic rats, was investigated in 2-month-old transgenic rats carrying human wild-type torsinA (tghwtTorAl11 rats) and non-transgenic animals as controls. The tghwtTorAl11 rats showed no NE-accumulation of torsinA but a few punctate structures (e). There was neither torsinA accumulation nor torsinA inclusion body in non-transgenic control (f). Scale bar: 200 µm; magnified inset, scale bar: 10 µm. (B) Co-immunofluorescence staining with anti-NeuN (red) and anti-torsinA (green) showed the NE-accumulation of torsinA only in NeuN positive cells (arrowheads). Scale bar: 10 µm.

The NE-accumulation of mutant torsinA is a characteristic neuropathological feature of DYT1 which shows strong torsinA immunoreactivity in a circle surrounding the nucleus in neuronal cells (Gonzalez-Alegre and Paulson, 2004; Goodchild and Dauer, 2004; Grundmann et al., 2012, 2007). NE-accumulation of mutant torsinA was found in tghΔGAGTorAl8 rats starting at P0 in the brainstem, where the highest protein expression level of the transgene was detected. At P14 the accumulation was also visible in the striatum and the hippocampus (data not shown). At P60, the NE-accumulation of torsinA could be detected in most brain regions including the striatum, hippocampus and cerebral cortex (Fig. 3A,a-c). Notably, the accumulation of torsinA in the NE seemed to disappear in the brainstem with decreased protein expression level (Fig. 3A,d). In the cortex, NE-accumulation of torsinA was more prominent in layers II-III where the protein expression was found to be increased drastically, while NE-accumulation was barely detected in layers IV-V (Fig. 3A,b). The most intense accumulation in the hippocampus was found in the DG and CA1 zone as reported for tghΔGAGTorAl4 rats (Grundmann et al., 2012). In contrast, cells in the CA3 zone showed only slight NE-accumulation and CA2 showed no NE-accumulation of torsinA at all in the analyzed time window (P0-P60). Few cells with NE-accumulation were found in the molecular layer of hippocampus (Fig. 3A,c). In the cerebellum, very high intensity of torsinA staining was present in the Purkinje and granular cells, however NE-accumulation was not detected in both cell types (Fig. 3A,d). We also investigated the striatum, the brain area with the most abundant NE-accumulation of torsinA, in the age-matched transgenic rats carrying human wild-type torsinA (tghwtTorAl11 rats) and non-transgenic animals as controls. NE-accumulation was neither found in the human wild-type torsinA controls, nor in the non-transgenic controls (Fig. 3A,e,f).

Moreover, we identified inclusion bodies in different brain regions, which is another well-known feature of torsinA pathology. However, cellular localization of inclusion bodies was different in selective brain regions including the striatum, cortex and hippocampus. In the striatum, inclusion bodies appeared around the nucleus, while in the cerebral cortex these structures were evident in the cytoplasm and proximate dendrites. In the hippocampus, inclusion bodies were found in both the neuropil and around the nucleus [Fig. 3A,a-c (insets)]. The tghwtTorAl11 rats expressing wild-type human torsinA exhibited a few punctuate structures similar to the inclusions observed in tghΔGAGTorAl8 rats carrying human mutant torsinA (Fig. 3A,e). No deposition of torsinA was found in non-transgenic animals at 2 months of age (Fig. 3A,f).

To determine the selectivity of cell populations which exhibited inclusion bodies, double immunofluorescence staining of NeuN and torsinA was performed on brain sections of tghΔGAGTorAl8 rats at 2 months of age. TorsinA NE-accumulation and formation of inclusion bodies appeared to be specific to NeuN-positive cells (Fig. 3B).

### NE abnormalities started at P0 in tghΔGAGTorAl8 rats

**Fig. 4.**
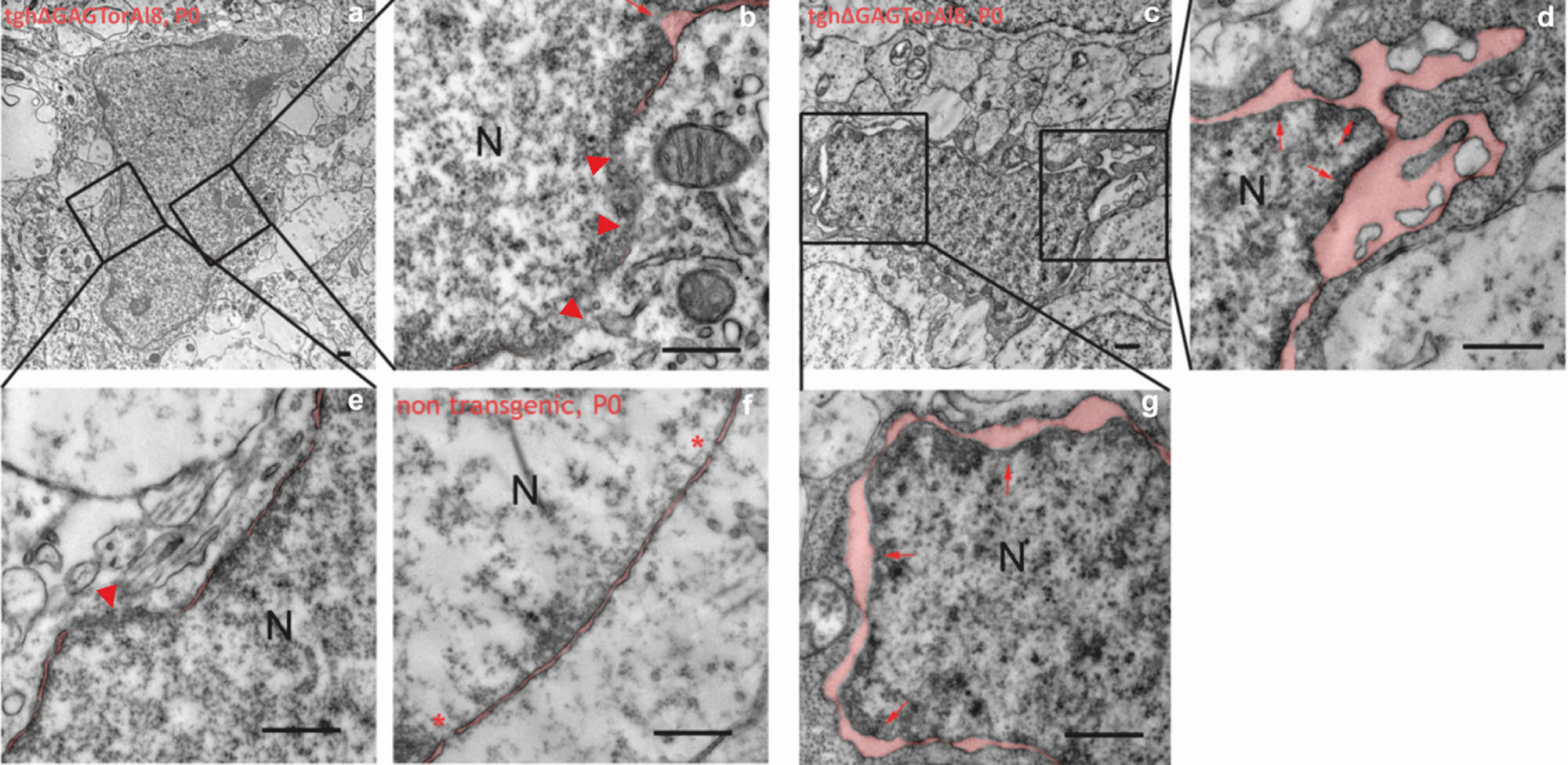
**Electron microscopy pictures of striatal neurons showed that pathological alteration was already present at birth.** Electron microscopy pictures of the striatal neurons (A,C) of newborn transgenic rats (line 8) showed pathological abnormalities in the NE such as discontinuous NE membranes (arrowheads, B,E) and enlarged perinuclear space of the NE (red arrows, B,D,G) in contrast to non-transgenic striatal neurons (F). Scale bars: 0.5 µm. Red asterisks indicate the nucelar pore of the NE in non-transgenic striatal neurons. N, nucleus. For better visualization, the perinuclear space is highlighted with bright red color.

As it has been reported that mutant torsinA affected the ultrastructure of the NE, we analyzed the NE membrane structure of striatal neurons utilizing electron microscopy at the age of P0 in tghΔGAGTorAl8 rats and controls. Our findings showed a discontinuous NE membrane and an enlarged perinuclear space in the striatum. Non-transgenic control rats showed a regular NE structure and a double membrane with normal perinuclear space (Fig. 4).

**Fig. 5.**
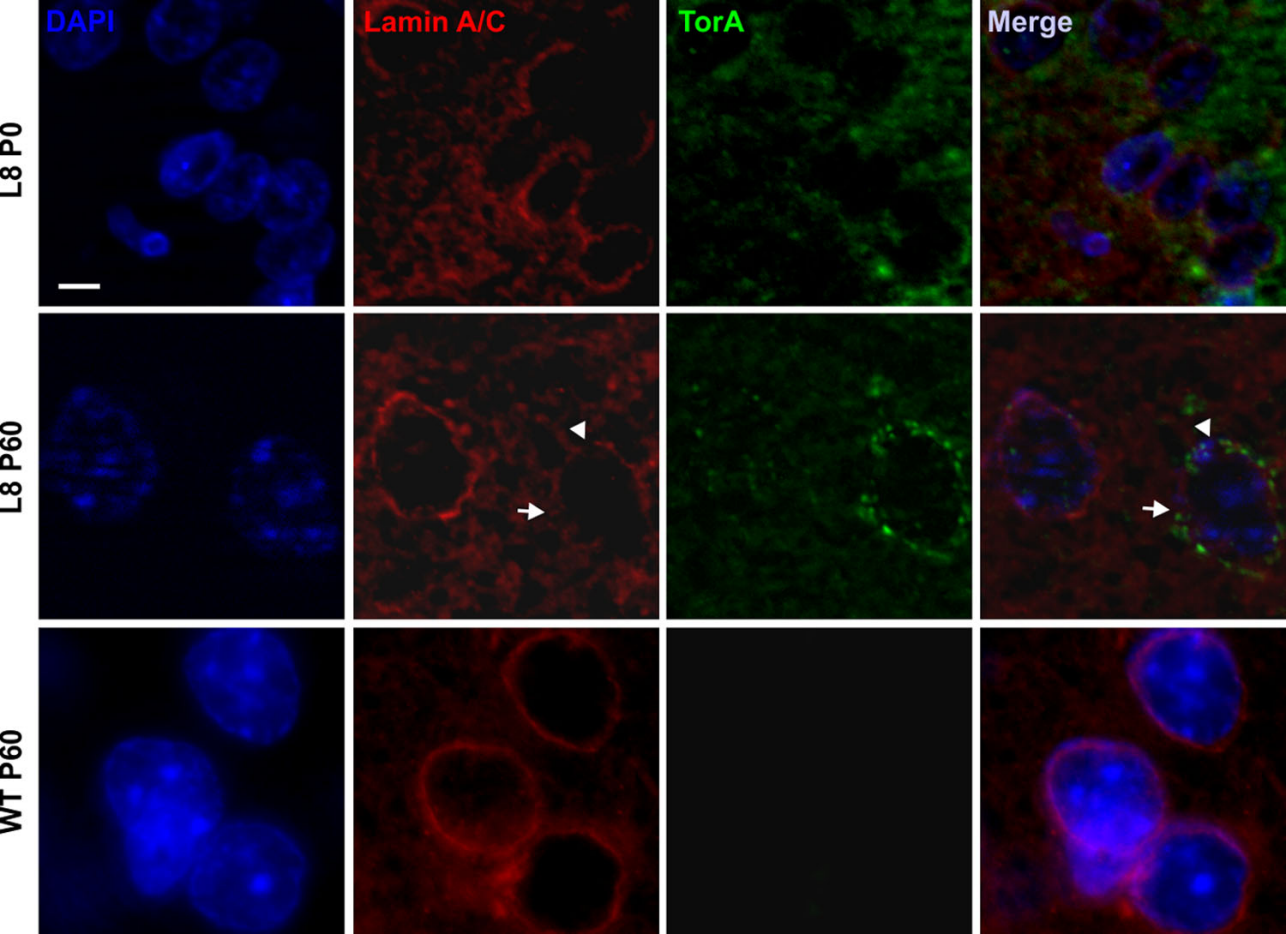
**Nuclear membrane abnormalities in tghΔGAGTorAl8 rats at P0 and P60 seemed to be associated with NE-accumulation of torsinA.** Brain sections of rats at P0 and P60 were stained with DAPI (blue), anti-lamin A/C (red) and anti-torsinA (green). Neurons of P60 tghΔGAGTorAl8 rats with NE-accumulation of torsinA showed a more prominent loss of nuclear membrane integrity (arrowheads) and abnormal membrane structure (arrows) in comparison to neurons of the same animal lacking NE-accumulation of torsinA, as well as neurons of tghΔGAGTorAl8 rats at P0. Non-transgenic animals of the same age showed a continuous normal structure of the nuclear membrane. Scale bar: 5 µm.

To further analyze the alteration of NE membrane organization and its association with torsinA accumulation, double immunofluorescence staining with anti-lamin A/C and anti-torsinA was performed on the brain sections of tghΔGAGTorAl8 rats at P0 and P60, when torsinA accumulation has started and reached an abundant level. We observed that the integrity of laminA-containing structures was unaltered at P0; however, at P60 a discontinuous laminA staining was clearly evident using immunofluorescence staining, showing diffuse lamin A/C immunoreactivity at some areas of the NE. These abnormal laminA-positive structures with moderate immunoreactivity of lamin A/C were only present in cells with NE-accumulation of torsinA, while torsinA accumulation-free cells showed a continuous structure of the NE with stronger lamin A/C staining, similar to wild-type littermates at P60 (Fig. 5).

### Mutant torsinA shifted from cell soma to neuropil in advanced age in tghΔGAGTorAl8 rats

**Fig. 6.**
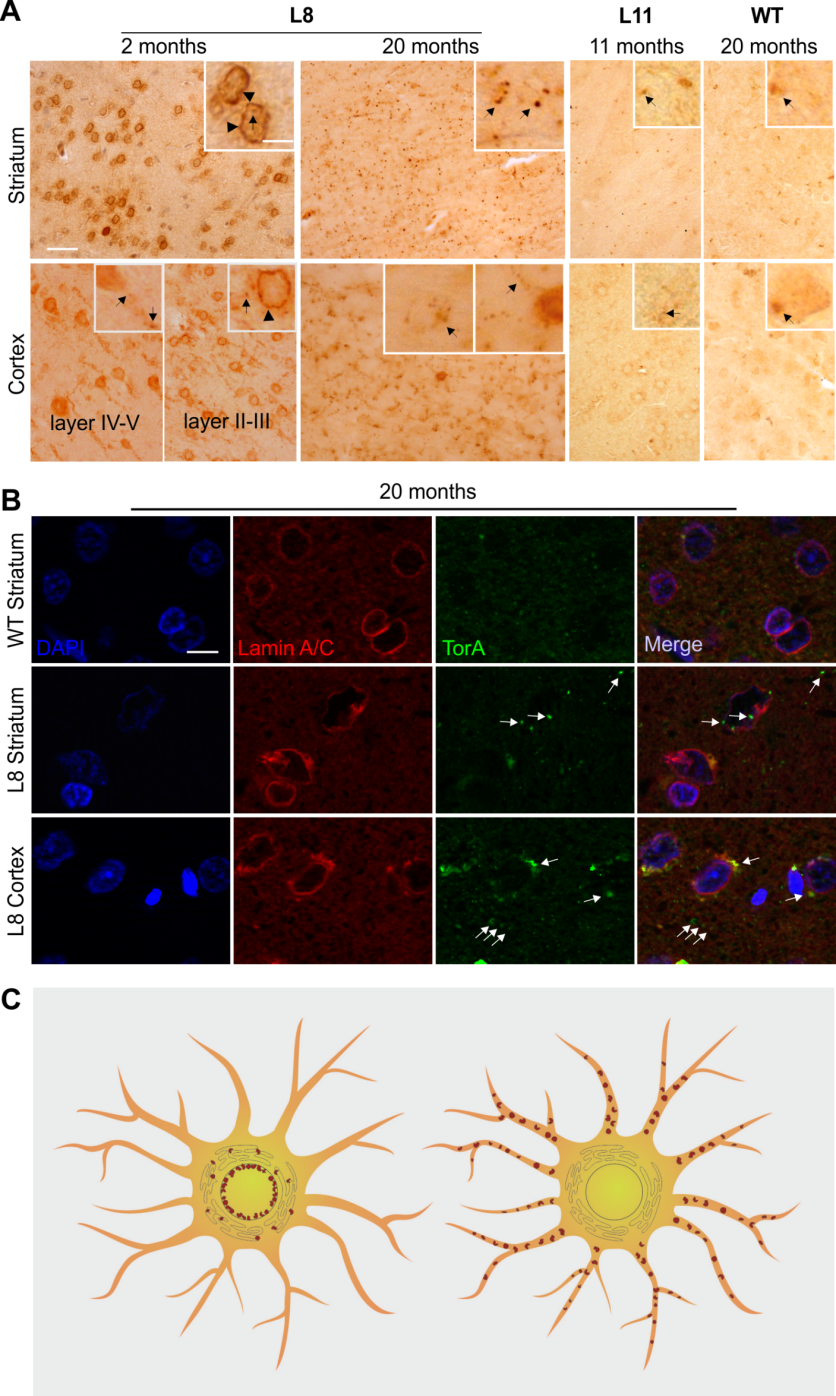
**Subcellular distribution of NE-accumulation and inclusion bodies of torsinA at younger and older ages in tghΔGAGTorAl8 rats.** (A) Brain sections of tghΔGAGTorAl8 rats at 2 and 20 months were stained by anti-torsinA. In comparison with sections from younger rats, the NE-accumulation (arrowheads) of torsinA was not apparent at 20 months of age, while the abundance and especially the size of punctate torsinA-positive structures (inclusion bodies, arrows) in the striatum were dramatically increased. The striatum of tghwtTorAl11 rats expressing human wild-type torsinA showed no NE-accumulation, but punctate structures that were similar to the inclusion bodies observed in the tghΔGAGTorAl8 rats had much reduced abundance (age of 11 months), while the cortex exhibited diffused cytoplasmic staining of torsinA with a few punctate structures. Both the striatum and cortex of non-transgenic rats at the age of 20 months only showed condensed punctate staining with different size and shape of inclusion bodies in the transgenic rats. Scale bar: 50 µm. Scale bar in magnified inset: 10 µm. (B) Subcellular localization of torsinA inclusion bodies in the striatum and the cerebral cortex at 20 months of age investigated by co-staining with DAPI (blue), anti-lamin A/C (red) and anti-torsinA (green). In the striatum, torsinA inclusion bodies (arrows) were present inside and outside the nucleus, and very few of them co-localized with NE, while most inclusion bodies exhibited at the cytoplasmic level in the cortex. TghΔGAGTorAl8 rats showed a restored nuclear membrane integrity at 20 months of age. Scale bar: 10 µm. (C) Scheme showing the dynamic localization of mutant torsinA protein in transgenic rats in distinct neurons of the striatum, cortex and hippocampus. At the age of 2 months (left) torsinA-containing structures are predominantly localized in the NE. At the age of 20 months (right) the torsinA punctate staining disappeared from the NE and is predominantly found in neuropils or the cytoplasm.

Interestingly, a different subcellular localization pattern of torsinA was demonstrated by immunohistochemical staining at the age of 2 months (Fig. 6A). Higher magnification revealed a ring-shaped torsinA signal in distinct neurons of the striatum and the cortical layers II and III (Fig. 6A) in contrast to diffuse cytoplasmic staining in neurons of the cortical layers IV and V.

Surprisingly, at the age of 20 months, the ring-shaped torsinA NE-accumulation disappeared and was replaced by diffused punctate structures in the striatum, cortex and other brain regions, indicating a cellular shift of torsinA signals from the NE to the neurites of cells in the tghΔGAGTorA rat brains. The abundance of punctate torsinA positive structures correlated with the severity of NE-accumulation and was most prominent in the striatum at 2 months of age. Relatively fewer punctate structures were found in other brain regions such as the cortex. There were no differences in the size and abundance of punctate structures, or in the distribution pattern, between cortical layers II-III and layers IV-V. We also investigated protein deposition in human wild-type torsinA-expressing rats and non-transgenic controls. The striatum of tghwtTorAl11 rats that expressed human wild-type torsinA showed no NE-accumulation, but did show punctate torsinA-positive structures with increased abundance at the ages of 2, 5, 8 and 11 months (data were only shown at the age of 11 months). Diffused cytoplasmic staining of torsinA, together with a few small punctate torsinA-positive structures, were found in the cerebral cortex of tghwtTorAl11 rats. Consistent with the ER-associated staining, both striatum and cortex of age-matched non-transgenic rats only exhibited condensed punctate staining, in contrast to the punctate torsinA-positive structures detected in the transgenic rats in terms of their size and shape (Fig. 6A).

We showed earlier that the integrity of the NE membrane correlated to the NE-accumulation of torsinA in rats at 2 months of age. Since the NE-accumulation seemed to have disappeared and was not evident at 16 months of age, this raised the question of whether NE integrity could be restored. We therefore performed co-immunofluorescence staining, using anti-lamin A/C and anti-torsinA, on brain sections of tghΔGAGTorAl8 rats at 20 months of age. In the striatum, small punctate structures were mainly detected in dendrites. Interestingly, cell soma did not exhibit larger punctate structures and lamin A-positive structures in the NE appeared to be well preserved. In the cortex, these punctate structures were found in cell bodies that remained connected to the outer nuclear membrane and consisted of at least lamin A/C and torsinA, as indicated by the double immunofluorescence labeling. Lamin A/C-containing structures appeared distorted but were relatively improved when compared with earlier time points (Figs 3A,b, 5 and 6B).

## DISCUSSION

Dystonia is a complex condition and even when the genetic cause has been disclosed for many years, as it has been the case for DYT1 dystonia, the underlying mechanism of the pathophysiology remains unclear. Despite the fact that several mouse models have already been generated for DYT1 dystonia, findings are inconsistent and the role(s) of mutant torsinA in the development of DYT1 dystonia and the physiological function(s) of this protein in its active or native state are still not well understood (Dang et al., 2005; Goodchild and Dauer, 2004; Goodchild et al., 2005; Grundmann et al., 2007; Sharma et al., 2005; Shashidharan et al., 2005). Based on current literature, we are not able to distinguish between the dystonia-like features observed *in vivo* and *in vitro* that are caused by torsinA dysfunction and those that are due to the bias of the animal model used. The findings from animal models so far have only partially recapitulated human conditions and have pointed towards biological processes that are not associated with human dystonia, such as the NE pathology and the inclusions. It is therefore important that a model system that can reproduce the torsinA expression patterns and disease-related phenotypes is available to address fundamental questions of disease mechanism and development.

To address this, we have generated a rat model over-expressing the mutant human torsinA gene under the control of the human *TOR1A* promoter. The transgenic rats, published as line 4 (Grundmann et al., 2012), showed a motor deficit, an alteration of synaptic plasticity and a cellular phenotype, all of which are dystonia-like characteristics as observed in other models and in patients. However, this line developed a reduced glucose tolerance at older ages. Hence, in order to analyze the later stages of the disease development and to reduce unwanted burden for the animals, we have characterized a second transgenic rat line that lacks the diabetes phenotype in this study. We have adopted the same approach as in Grundmann et al. (2012) to characterize the second rat line that expresses the human mutant torsinA under the control of the human promoter as well as the regulatory elements so as to mimic the human expression patterns of torsinA. Longitudinal behavioral assessments and neuropathological analyses were performed in order to correlate the key pathological features at cellular level to the observed behavioral abnormalities.

### Progressive motor impairment

Motor behavioral tests of line 8 revealed a similar phenotype as described in line 4 (Grundmann et al., 2012). Both human mutant torsinA-expressing rat lines reached significantly higher scores (i.e. more severe impairment) that were progressive over time in the clasping test, indicating motor deficits that mainly affected the hind limbs. In addition, these rats showed a significantly delayed learning behavior in a motor learning task as demonstrated by the second training session of the rotarod test when compared to line 4 or wild-type rats. Similar to line 4 rats, animals of line 8 showed normal behavior in the PhenoMaster home cages up to an age of 11 months. Together with our previous findings (Grundmann et al., 2012) that both lines are showing a mild and progressive motor impairment, we could conclude that these dystonia-like features are mutation-specific outcomes rather than the results of the model system chosen.

### Age-dependent and regional specific changes of torsinA intracellular expression patterns in different brain regions

Immunohistochemical analyses using monoclonal antibody D-M2A8 specifically recognizing human torsinA showed that the expression patterns of mutant torsinA in the brains of lines 4 and 8 are highly similar. High expression levels of cytoplasmic and potentially ER-associated intracellular torsinA were detected in the striatum, cortex, cerebellum and hippocampus and were found to be age-dependent. At P0, abundant protein expression was found in the brainstem, the cerebellum and the hippocampus, and less abundant mutant torsinA protein was present in layers IV-VI of the cerebral cortex, the olfactory bulb and the hypothalamus. Interestingly, from P0 to P60, the protein expression level of mutant torsinA increased in most brain areas, especially in the striatum and the cortical layers II-III, while it decreased in the cortical layers IV-VI and the brainstem. This demonstrated that expressing torsinA under its endogenous promotor could closely mimic the dynamic temporal and spatial expression pattern as observed in non-transgenic animals and humans, which is also consistent with our previous report (Grundmann et al., 2012). However, the results of the immunohistochemical analysis of the expression pattern have to be interpreted with care, since we were not able to exclude that the expression of human torsinA has affected the expression of endogenous rat torsinA.

In accordance with previous findings (Gonzalez-Alegre and Paulson, 2004; Goodchild et al., 2005; Naismith et al., 2004; Tanabe et al., 2009, 2016), we have also demonstrated in the present study that the ultrastructure of the NE was severely altered, which is a characteristic neuropathological feature of mutant torsinA, using immunofluorescence and electron microscopy and samples taken from various brain regions and ages of mutant torsinA-expressing transgenic rats.

When we analyzed mutant torsinA expression on the cellular level, we noticed a dynamic expression pattern of torsinA. NE-accumulation of mutant torsinA was detected already at P0 in the brainstem, where the highest expression level of the transgene was observed, while in the striatum, mutant torsinA expression was not evident before P14, indicating that NE-accumulation was directly correlated to the expression level of the mutant torsinA protein. Moreover, accumulation of mutant torsinA in the NE disappeared with decreasing protein expression levels, as shown in the brainstem. Instead, with decreasing expression levels, more granule torsinA-positive structures (inclusion bodies) were found in the proximity of the NE and neurites.

In the hippocampus and cortex, we found strong NE-accumulation of torsinA that were already present at P0, and a massive change of intracellular expression patterns with a shift of torsinA from NE to neurites in older rats (12-20 months), and they were mainly seen in pyramidal cells of cortical layers and hippocampal formation. The same NE-accumulation of torsinA was detected in the striatum with statistically the highest number of NE pathological neurons, however, the subsequent shift of torsinA from NE membrane to neurites was not observed to the same extent as in the cortex and hippocampus. To summarize, the cellular expression pattern of torsinA changes over time. Initially, torsinA accumulates in the NE membrane but is later shifted into the periphery and is found, in later stages, in neuropil of axons and dendrites. The integrity of the NE seems to be tightly associated to torsinA expression in neurons, since we were able to detect cells expressing torsinA next to cells that were not having visible torsinA expression. However, a discontinuous NE membrane structure determined by means of lamin A/C immunoreactivity was exclusively present in cells with NE-accumulation of torsinA, while torsinA accumulation-free cells showed a continuous structure of the NE with stronger lamin A/C staining similar to wild-type littermates at P60. In cells expressing torsinA, NE structure was restored as soon as torsinA was shifted into the cell periphery.

These data clearly demonstrate that disintegration of structures within the NE happens in cells expressing mutant torsinA and that these structures can be restored during further maturation of neurons (Tanabe et al., 2016).

Since the motor phenotype and molecular ultrastructural key features of torsinA pathology can be replicated, the characterized transgenic rat line 8 expressing human mutated torsinA was identified to serve as an appropriate model system for future investigations concerning therapeutic approaches and to be used as a powerful tool to assess disease development studies. Moreover, by analyzing the torsinA expression pattern in different brain regions at different points in time, we provided further evidence that the distorted laminA/C NE structure is not a persistent phenomenon but happens in a certain time window during brain development and is different for cell types in different brain regions. This points to a cell subtype-specific vulnerability, which is likely to be based on biochemical differences between neuronal subtypes of distinct brain regions. Whether this pathology is caused by torsinB as reported by Tanabe et al. (2016) or is influenced by other modifying factors remains to be investigated.

To summarize, in the present study, we have provided a model system harboring the potential of revealing the native function(s) of torsinA. Nevertheless, this model system also contains limitations such as integration site of the construct, copy number variances and interaction with the rat genome. Also we would like to highlight the fact that only over-expressing the human full-length construct is not enough to fully mimic the human condition.

## MATERIALS AND METHODS

### Animal housing

All animals were housed (when not used in experiments) under standard conditions with 12 h light/dark cycle and food and water *ad libitum*. The animal facility kept the temperature at 22±2°C, relative humidity at 55±10%, and was set to a partially inverted light/dark cycle with lights on/off at 02:00/14:00 during summer, and 01:00 h/13:00 h during winter. All tests were approved by the local ethics committee (Regierungspraesidium Tuebingen) and carried out in accordance with the German Animal Welfare Act and the guidelines of the Federation of European Laboratory Animal Science Associations based on European Union legislation (Directive 2010/63/EU).

All animals were of Sprague-Dawley background. Animals were genotyped according to previously published protocols (Grundmann et al., 2012) and housed in groups of three to four in type IV cages (38×55 cm) with high lids (24.5 cm from cage floor). All behavioral experiments were performed with a cohort of male rats, *n*=45 rats (15 tghΔGAGTorAl4, 15 tghΔGAGTorAl8 and 15 wild-type littermates rats chosen equally from both lines) at the age of 2 and 11 months by a trained observer that was blind to the genotypes. All behavioral tests (except the PhenoMaster test) were performed during the dark phase. Spontaneous activity and feeding as well as drinking behavior were measured in the PhenoMaster system over a period of 72 h, including three light/dark cycles.

### Hind limb clasping

To assess the hind limb clasping reflex as a marker of disease progression, rats were suspended by their tails and their behaviors were recorded for 10 s at 2 and 11 months of age. Hind limb clasping behavior was scored on a scale from 0 to 2 with the highest score given to the most severe phenotype. In more detail, the following scores were given: ‘hind limbs extended with spreading toes’=0 points, ‘hind limbs almost in contact with spreading toes’=1 point and ‘hind limbs clasped or crossed with toes flexed’=2 points. Each test was performed three times, and an average was taken for each rat. The sampling distribution of the clasping phenotype was analyzed with a chi-square-test; a non-randomness distribution of the clasping phenotype was reported to be significant at a 5% level.

### Rotarod test

Motor coordination and learning ability in tghΔGAGTorAl4, tghΔGAGTorAl8 rats and non-transgenic littermates were assessed using the accelerating rotarod test at 2 and 11 months of age. Rats were trained for three consecutive days with two sessions per day, followed by two consecutive testing days with two tests per day. During the first three training days, the rats were trained to a stable performance. At this stage, the maximum rotation speed of the rod was set to 12 rpm (increasing from 2 to 12 rpm over 30 s), and trials lasted 120 s. During these 120 s, rats were replaced onto the rod after falling, and the number of falls was recorded. The number of falls served as the performance readout. On the last 2 days, the rats’ maximum motor capacity was tested. For this, the rotation speed of the rod increased from 4-40 rpm over 240 s and the trials lasted either 300 s or until the rat had fallen off the rod, and the latency to fall was recorded.

### Beam walking test

The beam walking test was used as a second test to evaluate motor coordination and balance in rats at 2 and 11 months of age. During the beam walking test, the rats had to traverse a series of three different beams to reach a safety platform. The beams consisted of 2 m long wood strips with a square cross-section of 3 cm×3 cm, a rectangular cross-section of 2 cm×4 cm, or a round cross-section with diameter of 3.5 cm. The beams were located 70 cm above the ground. Training sessions were performed with three tests per session, followed by a single test session per beam. During the training days, the rats had to traverse the square cross-beam three times. On testing days, the rats had to traverse each beam once, starting with the square cross-sectioned beam, followed by the rectangular and finally the round cross-sectioned beam. Beam traversal time was recorded. Rats that did not cross the beam from the starting platform to the stop platform were excluded from analysis.

### PhenoMaster test

The PhenoMaster system (TSE Systems, Bad Homburg, Germany) is an automated cage system that allows behavioral and metabolic monitoring, e.g. activity, food and water consumption, as well as calorimetric measurements. The system consists of a combination of sensitive feeding and drinking sensors for automated online measurements. Values for cumulative amounts of feeding and drinking were given by the system and these were used to compare iterative food and water intake. The calorimetric system is an open-circuit system that determines O_2_-consumption, CO_2_-production, and respiratory exchange ratio (RER). To investigate locomotor activity, a photo beam-based activity monitoring system detects and records the number and duration of every total, fine, and ambulatory movement including rearing (Urbach et al., 2014). See Grundmann et al. (2012) for detailed description of these experiments.

### Immunohistochemistry

Protein expression level and subcellular localization as well as aggregation of mutant torsinA were investigated at P0, P60 and 20 months of age (three animals per group at each time point) by immunohistochemistry. Rats were deeply anesthetized with ketamine/xylazine (100/10 mg/kg, i.p.) and transcardially perfused with 4% paraformaldehyde in PBS buffer, pH 7.4, followed by post-fixation of the brains in the same fixative overnight. Brains were cut into 7 μm-thick sagittal sections using a Leica Instruments Microtome. Immunohistochemistry was performed as described previously (Grundmann et al., 2007) using torsinA monoclonal antibody D-M2A8 (1:100, mAb#2150 from Cell Signaling Technology), which specifically binds to human torsinA only (Hewett et al., 2003). Immunolabeling was visualized by biotin-coupled anti-mouse at the dilution of 1:250 (Vector Laboratories, Burlingame, USA).

### Immunofluorescence

To evaluate the subcellular distribution of accumulated torsinA and its selectivity to cell populations, double immunofluorescence staining using anti-torsinA (D-M2A8 1:100, Cell Signaling Technology) and anti-lamin A/C (1:100, sc-20681 from Santa Cruz Biotechnology), anti-torsinA and anti-NeuN (1:200, MAB377 from Millipore) were performed respectively. Paraffin brain sections were incubated at 4°C overnight simultaneously with primary antibodies. After subsequent washes, sections were incubated with the corresponding secondary antibodies (1:300, Dianova, Hamburg, Germany). After washing, sections were mounted with Vectashield mounting medium which contained DAPI for staining the nucleus (Vector Laboratories). Images were taken by an Axiofluor microscope and the imaging software Axiovision 4.9 (Carl Zeiss, Oberkochen, Germany).

### Electron microscopy

Brains of three transgenic male rats of tghΔGAGTorAl8 and three non-transgenic littermates at the age of P0 were dissected, immersed in Karnovsky's fixative and stored at 4°C. Brain tissues were washed in 0.1 M sodium cacodylate and post-fixed in 2% osmium tetroxide in 0.2 M sodium cacodylate. Tissues were dehydrated in a graded series of alcohol and then embedded in glycide ether and cut using an ultramicrotome (Ultracut, Reichert, Vienna, Austria). Ultra-thin sections (30 nm) were mounted on copper grids and analyzed using a Zeiss LIBRA 120 transmission electron microscope (Carl Zeiss).

### Statistical analysis

Statistical analysis was carried out using Statview and Prism statistical software package (GraphPad) as previously described (Grundmann et al., 2012).

All results are expressed as means±standard error. Results are reported as significant if *P*≤0.05. For multiple comparisons of data, one-way ANOVA or two-way ANOVA were used. Genetic status (tghΔGAGTorAl4, tghΔGAGTorAl8, non-transgenic control) was treated as between-subjects factor. Data were analyzed by one-way ANOVA or two-way ANOVA with Tukey or Dunnett post-hoc tests.

## Supplementary Material

Supplementary information
