# Supplementary material for: Dynamic nuclear envelope phenotype in rats overexpressing mutated human torsinA protein
Source: Biol Open. 2018 May 8;7(7):bio032839. doi: 10.1242/bio.032839 (PMC6078351; doi:10.1242/bio.032839)
Supplement: Supplementary information [file biolopen-7-032839-s1.pdf]

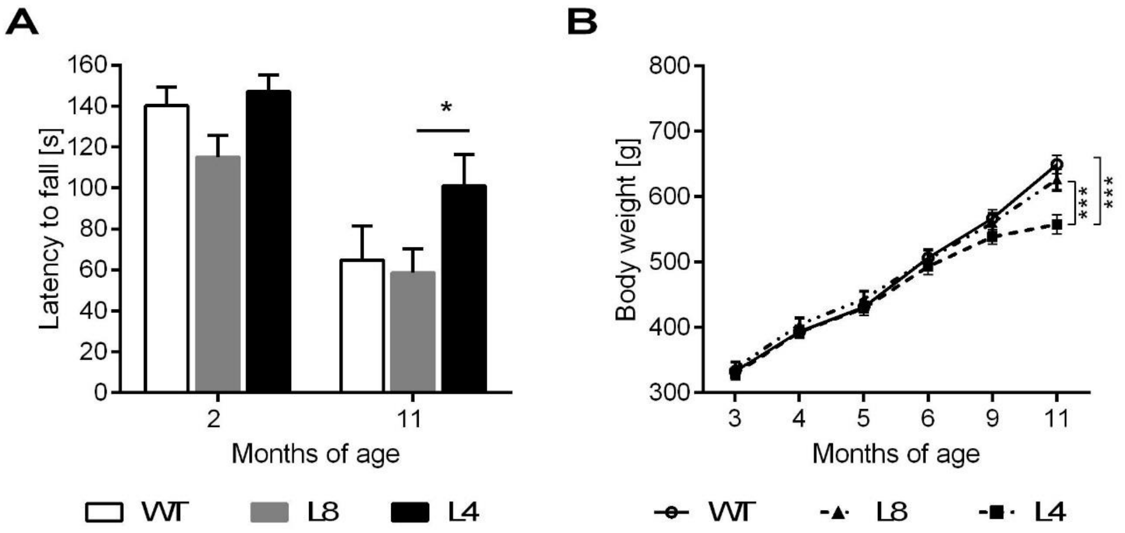


**Fig. S1. Rotarod performance and body weight.** A. Rotarod performance at 2 and 11 months of age. Only tghΔGAGTorAl8 rats at 11 months of age showed worse performance as compared to tghΔGAGTorAl4 rats. No difference was observed when comparing tghΔGAGTorAl8 rats to non-transgenic controls. B. Body weight from 3 to 11 months. Rats of line 8 showed a comparable body weight to non-transgenic controls over the investigation period, while rats of line 4 exhibited a strong reduction in body weight at 11 months of age. Data are presented as mean ± SEM. *: $p<0.05$; ***: $p<0.001$.

Biology Open • Supplementary information

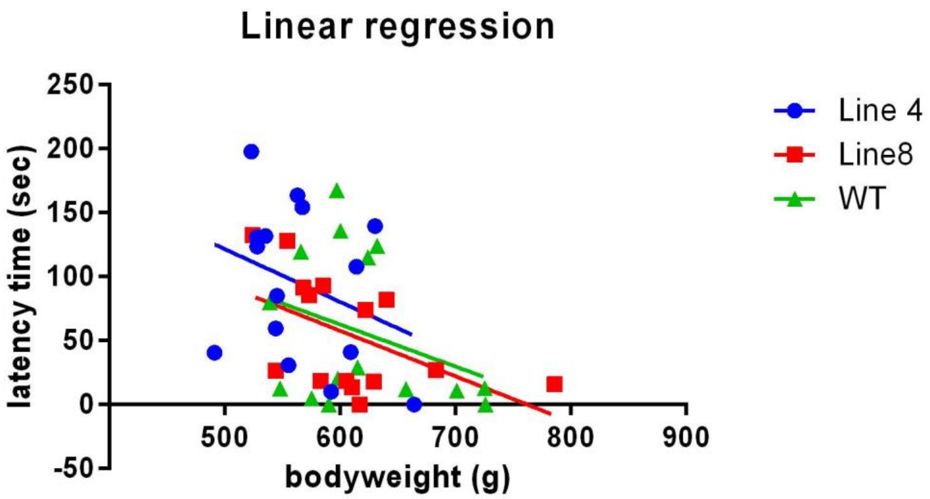


**Fig. S2. Linear regression analyses of the relation between body weight and the latency to fall.** Statistical analyses showed no significant differences in the slope of the curves between different genotypes (95% confidence intervals), suggesting a generally inverse correlation between the increased body weight and the latency to fall.

Biology Open • Supplementary information

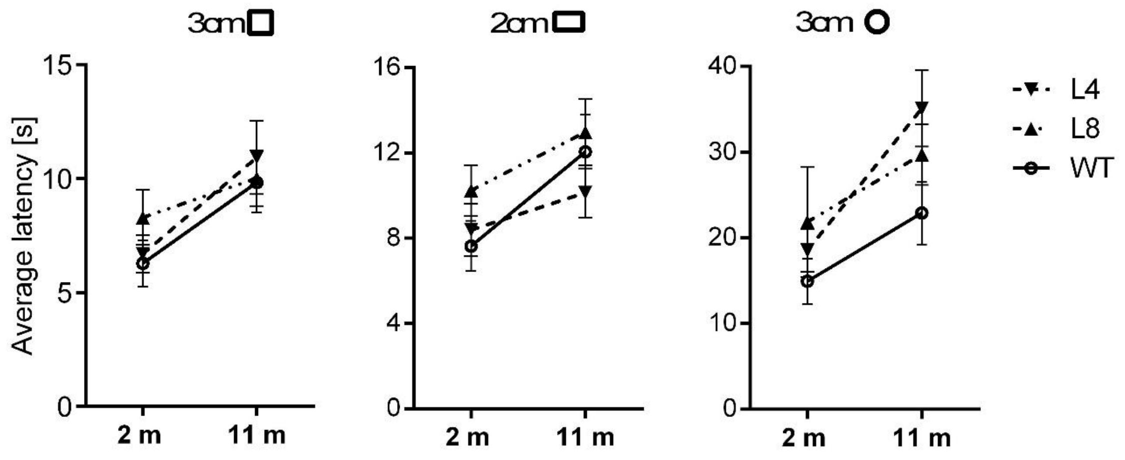


**Fig. S3. Beam walking performance.** On 2-meter-long beams with a square cross-section of 3 x 3 cm, a rectangular cross-section of 2 x 4 cm and a circular cross-section of 3 cm (diameter) (from left to right).

Biology Open • Supplementary information

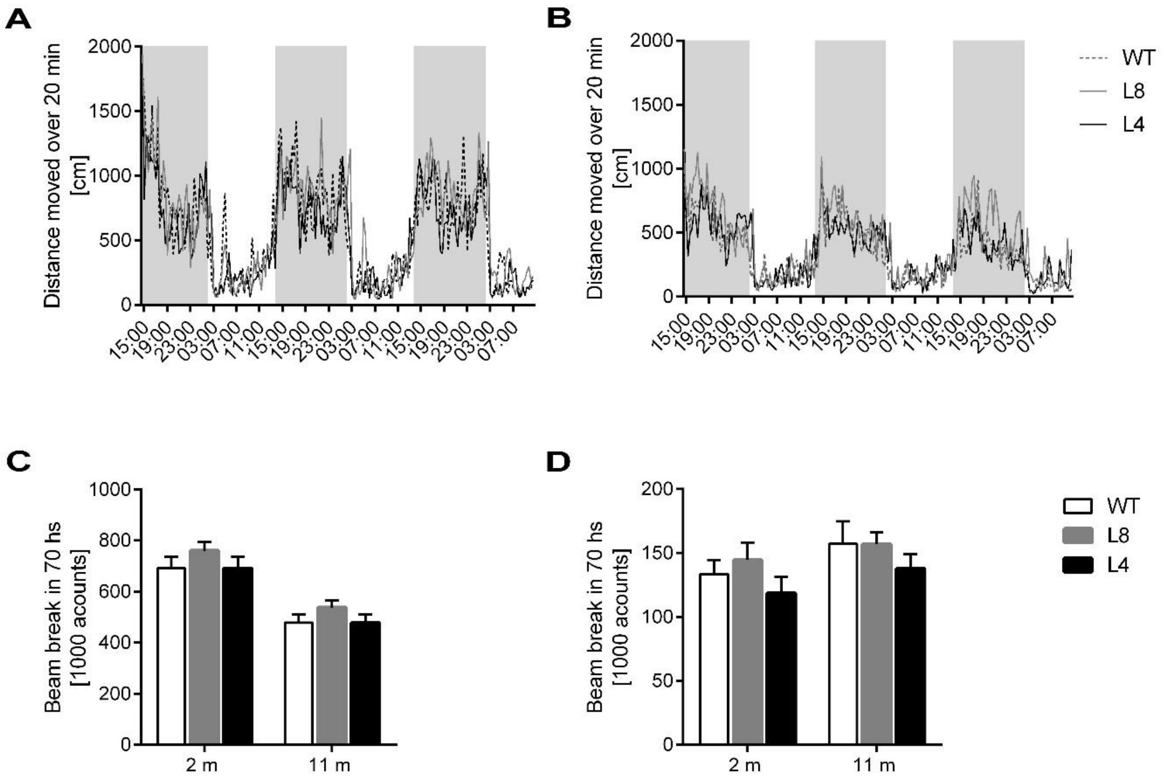


**Fig. S4. Measurements over 70 hours in PhenoMaster with a home cage-like environment.** A. Distance moved in every 20 minutes at the age of 2 months. B. Distance moved in every 20 minutes at the age of 11 months. C. Ambulatory activity (beam breaks on xy plane) during the dark phase at 2 and 11 months of age. D. Rearing activity (beam breaks on z plane) during the dark phase at 2 and 11 months of age.

Biology Open • Supplementary information
